# Phage Genomics and Bioinformatics in Therapeutic Development: Evidence Limits, Validation Gates, and Translational Decision-Making

**DOI:** 10.3390/ph19091465

**Published:** 2026-09-16

**Authors:** Mia Yang Ang, Li Chen, Lanni Song, Leonard Lipovich, Siew Woh Choo

**Affiliations:** 1 Department of Biomedical Sciences, Jeffrey Cheah Sunway Medical School, Faculty of Medical and Life Sciences, Sunway University, Sunway City, Petaling Jaya 47500, Selangor, Malaysia; 2 Sunway Microbiome Centre, Faculty of Medical and Life Sciences, Sunway University, Sunway City, Petaling Jaya 47500, Selangor, Malaysia; 3 Zhejiang-Malaysia Joint Laboratory for Rare Medicinal Resources, Wenzhou-Kean University, 88 Daxue Road, Ouhai, Wenzhou 325060, China; 4 College of Science, Mathematics and Technology, Wenzhou-Kean University, 88 Daxue Road, Ouhai, Wenzhou 325060, China; 5 Dorothy and George Hennings College of Science, Mathematics and Technology, Kean University, 1000 Morris Ave, Union, NJ 07083, USA; 6 International Frontier Interdisciplinary Research Institute (IFIRI), Wenzhou-Kean University, 88 Daxue Road, Ouhai, Wenzhou 325060, China

**Keywords:** phage genomics, bacteriophage therapy, antibacterial biopharmaceuticals, bioinformatics, phage-host prediction, genome-informed risk assessment, phage cocktails, phage-antibiotic combinations, product quality, regulatory science

## Abstract

**Background/Objectives:** Antimicrobial resistance has renewed interest in live therapeutic phages, including engineered candidates and defined phage cocktails, as antibacterial strategies. However, clinical translation requires evidence extending beyond phage isolation and computational prediction. This review examines how phage genomics and bioinformatics can support phage-based antibacterial development while remaining aligned with pharmaceutical requirements for safety, quality, pharmacology, and clinical validation. **Methods:** This narrative review synthesizes the literature on the generation, interpretation, and experimental validation of genomic and bioinformatic evidence for live therapeutic phages. Representative approaches for viral identification, genome-quality assessment, annotation, comparative genomics, lytic–temperate lifestyle classification, host prediction, receptor analysis, antiphage-defence profiling, and artificial-intelligence-assisted prioritization were evaluated according to their outputs, principal failure modes, validation requirements, and supported development decisions. **Results:** Current tools address distinct analytical tasks. Examples include VIBRANT and geNomad for viral identification, CheckV and PhageTerm for genome-quality and termini assessment, Pharokka, PHANOTATE, and PHROGs for gene prediction and annotation, PhageAI, BACPHLIP, and PhaTYP for lytic–temperate lifestyle prediction, iPHoP and CRISPR spacer matching for host prioritization, and PADLOC and DefenseFinder for bacterial defence profiling. Their outputs differ in taxonomic resolution, reference coverage, training-data dependence, and biological interpretation. The review maps these outputs to proportionate validation requirements while integrating formulation and PK/PD context, sequence-to-product traceability, intellectual-property documentation, minimum-information reporting, and resistance-responsive redesign. **Conclusions:** This review presents a practical sequence-to-product framework for interpreting contemporary phage-bioinformatics methods. By separating computational discovery from isolate-level activity, product quality, pharmacological evidence, and clinical monitoring, it clarifies the decisions supported at each stage and the additional evidence required before candidate progression, product use, or redesign.

## 1. Introduction

Antimicrobial resistance (AMR) remains a major global health challenge, with substantial mortality associated with bacterial AMR worldwide [1]. This burden has renewed interest in antibacterial strategies that can complement or extend beyond conventional small-molecule antibiotics. Bacteriophages are prominent in this discussion because they frequently exhibit narrow and strain-dependent host ranges, although specificity varies considerably among phages and bacterial populations [2].

However, phage-based antibacterial development is more complex than simply identifying a phage that can infect a bacterial isolate [3]. Modern therapeutic-phage development involves genome sequencing, comparative genomics, host-range assessment, genome-informed risk assessment, product-quality considerations, formulation, pharmacological interpretation, and regulatory expectations. Although phage therapy offers important opportunities, considerable heterogeneity remains across indications, product formats, and evidence standards. Challenges also remain in study design, product standardization, medicinal-product classification, and approval pathways [4].

Phage-based antibacterial strategies should therefore not be treated as a single therapeutic class [5]. Natural phages, phage cocktails, personalized preparations, engineered phages, and phage-antibiotic combinations each raise different questions about product identity, quality, risk, delivery, and clinical validation. This diversity has moved the field beyond classical phage therapy toward a broader antibacterial biopharmaceutical landscape, in which phage genomics and bioinformatics are increasingly used not only for discovery, but also to support candidate description, genome-informed risk assessment, host association assessment, product documentation, and translational decision-making [6].

### 1.1. Genomics and Bioinformatics as Translational Evidence

Genomics and bioinformatics are now central to the evaluation of phage-based antibacterial products [7]. Genome sequencing can support candidate identity, taxonomy, annotation, comparative analysis, and screening for risk-associated genomic features. Viral discovery tools have expanded the detection of phage and viral sequences in complex datasets, enabling the identification of previously uncharacterized phages from metagenomic and environmental sequencing data [8]. In parallel, viral-genome quality-assessment tools can estimate genome completeness and identify host-derived regions, while phage-focused annotation and nomenclature support the generation of reproducible and clearly described genome records [9,10].

The growing use of automated annotation, host-association prediction, lifestyle classification, comparative genomics, and artificial intelligence has substantially increased the number of phage candidates that can be screened before extensive laboratory characterization. However, these computational outputs do not have equivalent evidentiary meaning. A genome-completeness estimate, a predicted lytic lifestyle, a putative host assignment, and the apparent absence of risk-associated genes support different interpretations and require different forms of confirmation. The unresolved translational problem is therefore not simply whether computational predictions require experimental validation, but which orthogonal evidence is proportionate to each genomic finding and when the combined evidence is sufficient to support candidate selection, product characterization, or further development.

This distinction is becoming increasingly important in pharmaceutical and regulatory evaluation. In the United States, clinical investigation of CBER-regulated biological products is conducted through the Food and Drug Administration investigational new drug framework, which requires an integrated evidence package before an investigational product can be administered in clinical studies [11]. In Europe, the European Medicines Agency has developed a draft guideline specifically clarifying quality expectations for human phage therapy medicinal products, including manufacture, characterization, specifications, analytical controls, reference standards, pharmaceutical development, and stability [12]. These developments demonstrate increasing regulatory attention to phage products, but they also expose the need for clearer links between sequence-derived findings, experimental activity, manufacturing quality, stability, pharmacological evidence, and the identity of the product ultimately tested or administered.

Current reporting practices do not always make these evidentiary relationships explicit. Computational findings may be reported without clearly stating their uncertainty, the validation performed, or the development decision they are intended to support. Host-association predictions may be interpreted as evidence of susceptibility, lifestyle classifications may be treated as definitive evidence of obligately lytic behavior, and the absence of annotated risk-associated genes may be presented as proof of safety despite incomplete phage-protein annotation [13,14]. Such interpretations can weaken candidate-selection rationales and complicate comparison, reproducibility, product traceability, and regulatory review.

**Figure 1:**
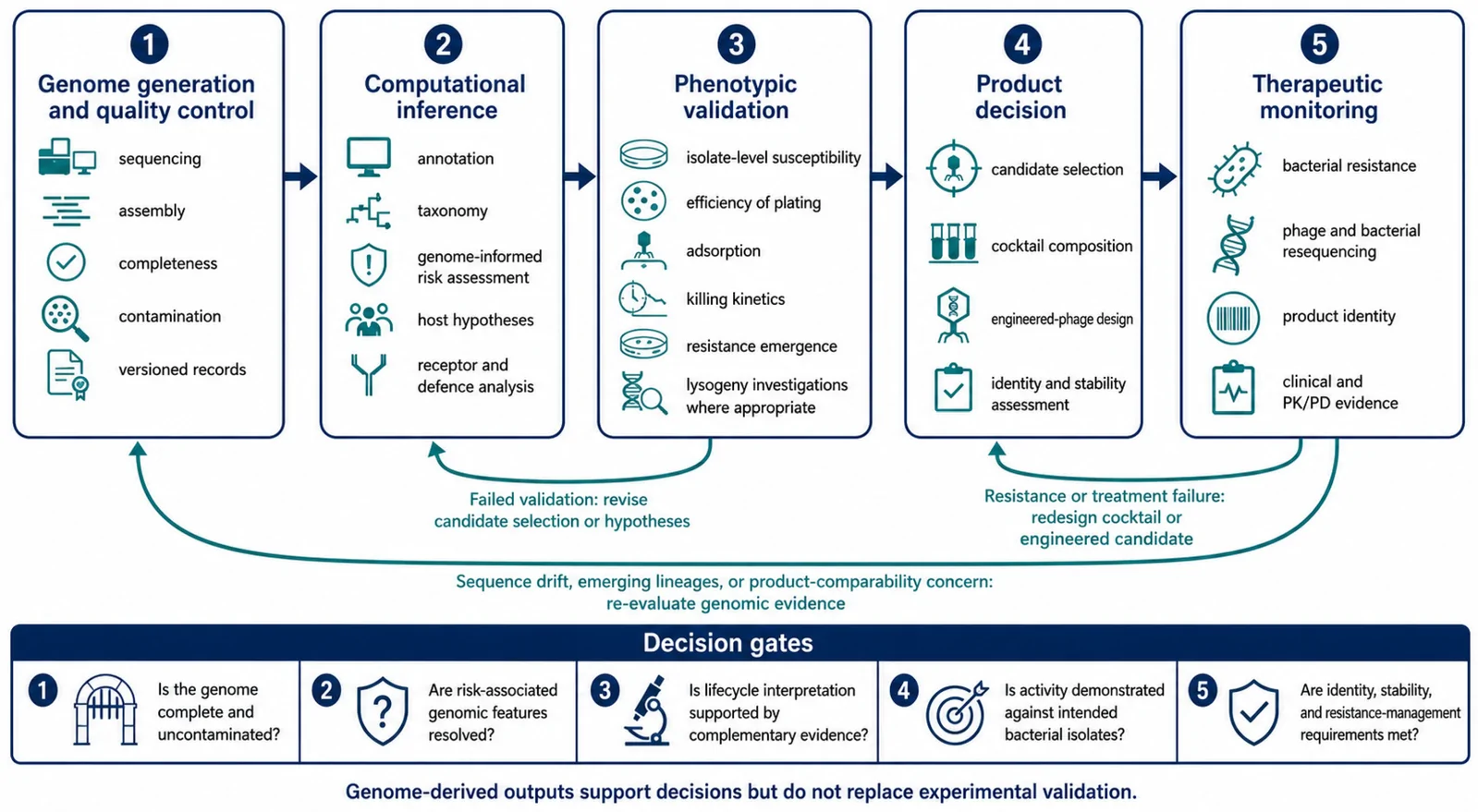
Iterative genome-informed framework for therapeutic-phage development. Genome generation and computational inference produce candidate hypotheses that must pass phenotypic and product-level validation gates. Therapeutic monitoring, resistance emergence, and product failure feedback to candidate selection, cocktail redesign, and engineering.

This review addresses this translational gap by examining how specific genomic and bioinformatic outputs can be connected to their principal failure modes, proportionate validation requirements, and supported development decisions. Recombinant phage-derived proteins are outside the principal scope because their computational, manufacturing, and pharmacological development pathways are more closely aligned with protein biologics. Unlike reviews centered primarily on phage biology, clinical use, or regulatory pathways, the present review frames phage genomics and bioinformatics as components of a traceable pharmaceutical evidence pathway. It builds on recent work on genomic analysis and standardization by emphasizing the validation gates between genomic inference, isolate-level susceptibility, product selection, quality assessment, therapeutic monitoring, and iterative redesign [15]. The framework links discovery, genomic characterization, computational interpretation, genome-informed risk assessment, product-selection rationale, quality evidence, and clinical validation (Figure 1). The central contribution is therefore not the general observation that computational predictions require validation, but a decision-oriented framework specifying what each computational output can support, where it may fail, which complementary evidence is required, and which subsequent therapeutic-phage development decision can be justified.

### 1.2. Scope and Narrative Review Approach

This narrative review focuses on live therapeutic phages, including unmodified lytic phages, defined and personalized phage cocktails, and engineered phages. Metagenomics, artificial intelligence, formulation, delivery, PK/PD, immune responses, regulatory considerations, sequence traceability, and intellectual property are discussed where they influence the interpretation, validation, or translational use of genomic evidence rather than as comprehensive reviews of these individual fields. Metagenomic sequences are treated as sources of candidate hypotheses until a physically defined phage is isolated or reconstructed and subjected to biological and product-level characterization. Recombinant phage-derived proteins, including endolysins and depolymerases, are not examined in detail because their manufacturing, quality-control, and pharmacological development pathways are more closely aligned with those of protein biologics [16].

The review was structured around four questions relevant to therapeutic-phage development: what a genomic or computational output can support; where that output may fail or be overinterpreted; which orthogonal, phenotypic, or product-level evidence is required; and which development decision can reasonably be supported after validation. This structure was used to distinguish candidate discovery and prioritization from isolate-level susceptibility, product identity, manufacturing quality, pharmacological interpretation, and clinical evidence. The purpose was not to rank software solely by computational performance, but to position representative tools and analytical strategies within the evidentiary pathway required for pharmaceutical development.

PubMed, Scopus, and Web of Science were searched for English-language literature published through July 2026 using combinations of terms related to bacteriophage therapy, phage genome sequencing, genome assembly, viral identification, genome-quality assessment, annotation, comparative genomics, Lytic–temperate lifestyle prediction, host prediction, receptor-binding proteins, CRISPR spacer matching, bacterial antiphage defence systems, artificial intelligence, phage engineering, phage cocktails, resistance monitoring, formulation, delivery, PK/PD, regulatory quality, sequence traceability, intellectual property, and minimum-information reporting standards. The narrative synthesis prioritised recent reviews, primary validation studies, clinical investigations, tool-development and benchmarking studies, regulatory documents, and reporting frameworks. Reference lists of key publications were also screened to identify additional relevant sources.

Recent reviews have described genome analysis, annotation, host prediction, clinical use, and genomic standardization in phage therapy, as summarized in Table 1. Rather than providing an exhaustive software benchmark or a general overview of phage therapy, this review integrates representative bioinformatics methods with their principal failure modes, required validation, pharmaceutical relevance, and supported development decisions.

The present review therefore focuses on a translational boundary that remains insufficiently explicit in broader reviews: genomic and computational outputs support different development decisions and require proportionate validation before they can be applied to patient-associated isolates, manufacturing control, or resistance-responsive product redesign. Genome analysis is accordingly treated as a series of decision-support layers, each linked to a principal failure mode, validation requirement, and supported development decision [5,7,18].

## 2. Phage-Based Antibacterial Biopharmaceuticals: Scientific and Translational Context

Phage-based antibacterial development sits at the intersection of microbiology, genomics, pharmaceutical science, and clinical translation. Although phages are often discussed in relation to antimicrobial resistance, their development as therapeutic products requires more than bacterial killing activity. A candidate must be identifiable, characterizable, supported by an appropriate product-specific risk assessment, compatible with an appropriate formulation, and supported by evidence that is relevant to the target pathogen and clinical indication [19].

This section introduces the scientific and translational context for phage-based antibacterial biopharmaceuticals. It first considers the role of phage therapy in the antimicrobial resistance landscape, then outlines the major product formats in current development, and finally summarizes the main translational barriers that influence the interpretation and integration of evidence across development stages.

### 2.1. Phage Therapy in the Antimicrobial Resistance Landscape

The first documented use of bacteriophages to treat infectious disease occurred more than a century ago [20]. Phage therapy as an alternative to antibiotics was extensively researched in the Soviet Union from the 1920s onward [21], but its adoption declined in many Western settings after the widespread introduction of antibiotics and because of persistent challenges related to standardization, clinical validation, intellectual-property protection, and commercial development [22].

Phage therapy has regained attention because bacteriophages can be selected against bacterial pathogens that are resistant to available antibiotics [17]. This makes them attractive for difficult-to-treat infections, especially when conventional treatment options are limited. However, their therapeutic value depends on successful matching to the target bacterium, acceptable quality, delivery to the relevant infection site, and appropriate clinical evidence [23].

The clinical evidence base remains promising but heterogeneous [24]. Published studies include case reports, compassionate-use experiences, observational studies, controlled trials, and early-phase product evaluations. These studies support continued investigation, but they do not establish uniform efficacy across pathogens, products, routes, or indications. Their interpretation is influenced by bacterial susceptibility, infection site, immune context, concomitant antibiotic therapy, product quality, route of administration, and clinical endpoints [25].

The antimicrobial resistance context therefore creates both opportunity and caution [26]. Phages offer a biologically plausible approach for targeting resistant bacteria, but resistance status alone does not define product suitability. A phage-based antibacterial product still requires a clear target, defined product identity, activity evidence, safety assessment, quality documentation, and clinical validation. Phage therapy should therefore be considered both a microbiological intervention and a pharmaceutical development challenge [4].

### 2.2. Product Formats in Phage-Based Antibacterial Development

Phage-based antibacterial development includes several product formats with different evidence requirements. Natural lytic phages remain the most familiar format, while fixed phage cocktails aim to broaden bacterial coverage through defined combinations. Personalized phage preparations are selected to match patient-associated bacterial isolates. Engineered phages, including CRISPR-based constructs, add further design possibilities, but they also raise additional questions about identity, stability, safety, intellectual property protection, and regulatory interpretation [5].

Product-format language is therefore not merely descriptive; it shapes the evidence required for development and evaluation. For live phages, genome identity, host range, infectivity, and genome-informed risk assessment are central. CRISPR-based phages are considered engineered phages rather than a parallel product format. For phage–antibiotic combinations, interaction evidence, resistance dynamics, and pharmacokinetic/pharmacodynamic (PK/PD) interpretation are central. A genomically suitable phage should therefore be viewed as a candidate component, rather than as a finished pharmaceutical product [27]. The major phage-based antibacterial product formats and their corresponding evidence requirements are summarized in Table 2.

### 2.3. Translational Barriers and Evidence Gaps

Clinical translation remains constrained by substantial heterogeneity in product composition, study design, treatment context, and outcome assessment [38]. Compassionate-use and expanded-access experiences demonstrate that individualized phage therapy can be feasible, but they also illustrate the difficulty of interpreting variable product composition, production documentation, treatment timing, concomitant therapies, and clinical response. Randomized and early-phase studies indicate increasing clinical maturity, but product-specific evidence remains necessary before efficacy or generalizability can be inferred across pathogens, products, routes of administration, or clinical indications [39].

Therapeutic development also faces evolutionary, pharmaceutical, and pharmacological barriers [40]. Bacteria can develop phage resistance through receptor modification, antiphage defence systems, altered physiological states, or other adaptive mechanisms. Phage–antibiotic interactions may be beneficial under some conditions, but they depend on the bacterial strain, phage, antibiotic, dose, sequence of administration, and exposure conditions. Phage pharmacokinetics and pharmacodynamics also differ fundamentally from those of conventional antibiotics because exposure may be influenced by bacterial density, local phage replication, immune clearance, formulation, delivery route, and access to the infection site [41].

The relevance of these barriers differs among product formats. Personalized preparations require rapid but traceable candidate selection and quality assessment. Fixed cocktails require evidence of component compatibility, combined host coverage, stability, and resistance escape. Engineered phages require confirmation of the intended genomic modification, genetic stability, retained activity, and evaluation of introduced functions. A product that performs well against one strain collection may not remain equally relevant across geographic regions, time periods, or bacterial lineages, while broad in vitro host coverage does not by itself establish product robustness or clinical utility [31].

Genomic and bioinformatic evidence should therefore be interpreted in relation to the product format, intended pathogen population, formulation, route of administration, and proposed clinical use. A computational result may support candidate prioritization or assay design without supporting a claim of product quality, pharmacological performance, or clinical efficacy. Translational interpretation consequently depends on linking genomic evidence with phenotypic activity, product quality, pharmaceutical development, PK/PD reasoning, and clinical context [42].

## 3. Biological Determinants and Pharmaceutical Modifiers of Phage-Based Antibacterial Development

Phage-based antibacterial development depends on both biological determinants of phage activity and pharmaceutical factors that influence whether this activity is retained at the intended infection site [43,44]. A candidate may appear suitable based on genome quality, lifestyle classification, or predicted host association, yet its translational value also depends on interaction with the target bacterium, stability during formulation and storage, delivery to the relevant bacterial population, and activity under clinically relevant conditions. This section therefore examines lytic activity, host range, receptor biology, resistance, formulation, delivery, PK/PD, engineered-phage concepts, and the remaining uncertainties that limit direct translation of genomic findings.

### 3.1. Lytic Activity, Host Range, Receptors, and Resistance

Therapeutic phage development commonly prioritizes lytic activity against the target bacterium [29]. Host range is equally important because a phage that is genomically attractive but inactive against the intended bacterial strain has limited therapeutic value. Host range is shaped by receptor availability, bacterial surface variation, antiphage defense systems, physiological state, and ecological context [45].

Phage adsorption begins with bacterial receptors, making receptor biology central to host specificity and resistance interpretation [45]. Bacteria may reduce phage susceptibility by modifying or losing surface receptors, activating antiphage defense systems, or changing physiological states. The consequences of these adaptations vary among systems. In some cases, phage resistance may reduce bacterial fitness or alter antibiotic susceptibility; in others, it may simply limit phage effectiveness [46].

Biofilm biology adds another layer of complexity [47]. Many difficult infections involve structured bacterial communities rather than planktonic cells alone. Biofilm architecture, matrix composition, capsule expression, nutrient gradients, and local bacterial physiology can influence phage access and activity. For this reason, antibiofilm claims should be linked clearly to the experimental model, readout, and evidence type used. Genome-based prediction, host-range testing, biofilm assays, and infection-site evidence should therefore be treated as complementary but non-interchangeable evidence.

### 3.2. Formulation, Delivery, and PK/PD as Translational Modifiers

Genomic suitability and demonstrated in vitro activity do not establish that a phage will retain activity after formulation, storage, administration, and delivery to the intended infection site. Product performance can be influenced by loss of infectivity during storage, formulation-dependent stability, interactions among cocktail components, delivery barriers, tissue or biofilm penetration, and exposure to local physiological and immune conditions [41,48,49,50]. These factors may differ substantially among oral, topical, inhaled, intravenous, and locally administered preparations and therefore affect how laboratory activity should be interpreted for a specific therapeutic product.

Phage PK/PD is also distinct from the exposure–response behaviour of conventional antibiotics. Therapeutic exposure may depend on the administered dose, adsorption to susceptible bacteria, local bacterial density, productive replication, spatial access to the target population, and clearance by the host immune system [41]. Consequently, the same phage may show different activity across planktonic cultures, biofilms, animal models, and human infection sites even when its genome and nominal host association remain unchanged.

Bioinformatics can support this pharmaceutical evidence pathway by confirming candidate identity, detecting sequence changes, comparing production or treatment-associated isolates, and maintaining traceability between the sequenced candidate and the formulated product. However, genomic analysis cannot determine formulation compatibility, retained infectivity, local exposure, release from a delivery system, or an effective dosing regimen. These properties require formulation-specific stability studies, potency testing, pharmacological assessment, and infection-relevant experimental models [48,50,51,52].

Formulation, delivery, and PK/PD should therefore be treated as modifiers of genomic and phenotypic evidence rather than as downstream considerations that become relevant only after candidate selection. Host-range predictions and in vitro killing results should be interpreted in relation to the intended route, infection site, formulation, and exposure conditions before they are used to support therapeutic-performance claims.

### 3.3. Engineered Phage Concepts

Engineered phage concepts further expand the design space. Genetic modification may be used to alter host range, introduce payloads, improve antibacterial activity, or support sequence-directed killing strategies such as CRISPR-enhanced concepts [53]. These approaches allow more defined design hypotheses, but each modification can also introduce additional uncertainties related to genome stability, safety, environmental considerations, and regulatory classification [54].

Genome engineering can pursue distinct, testable objectives. Receptor-binding proteins may be modified to alter host range, while undesirable functions may be deleted or inactivated [34,35]. Engineered phages may also be used to deliver antibacterial payloads or support CRISPR-based sequence-directed antibacterial activity [55,56,57]. Each objective requires sequence confirmation of the final construct, assessment of genetic stability, and product-specific tests for activity, escape, and unintended effects [34,57]. Computational design alone cannot establish therapeutic performance.

Engineering does not remove the need to characterize the resulting biological system. A modified receptor-binding protein may broaden adsorption in one strain panel but narrow activity in another; a payload or CRISPR construct may impose fitness costs, select escape variants, or alter host-range stability. Engineering studies should therefore report the parental and final genome sequences, genotype-confirmed edits, serial-passage stability, activity against independent clinical isolates, and resistance trajectories. These data are needed to connect design intent to a product-specific benefit–risk assessment rather than treating the engineered sequence as evidence of improved therapeutic performance [34,57].

### 3.4. Knowledge Gaps and Translational Uncertainty

Several unresolved biological uncertainties continue to limit the interpretation of genomic evidence across therapeutic-phage product formats [58]. Many phage proteins remain hypothetical or incompletely characterized, which reduces confidence in functional annotation, mechanistic interpretation, and genome-informed risk assessment. Apparent absence of a recognized risk-associated gene must therefore be interpreted in relation to genome quality, database coverage, annotation confidence, and the proportion of proteins with unknown function rather than treated as definitive evidence of suitability.

Strain-level activity also remains difficult to infer from genomic information. Receptor-associated features, sequence similarity, CRISPR spacer matches, and bacterial antiphage-defence profiles can strengthen mechanistic hypotheses, but infection outcomes depend on receptor expression, defence-system activity, bacterial physiological state, and interactions among these factors. Immune clearance and phage-specific immune responses may further alter exposure, repeat administration, and treatment monitoring [59]. These sources of variability explain why related phages or bacterial strains may produce different phenotypes despite apparently similar genomic features.

Uncertainty should therefore be reported according to the development question being addressed. Genome analysis may support candidate identity, functional hypotheses, risk-based prioritization, or assay design, whereas isolate-level susceptibility, product stability, pharmacological performance, and clinical activity require distinct evidence. The required validation should be proportionate to the consequence of the decision: preliminary candidate prioritization may tolerate greater uncertainty than exclusion of a candidate, selection of a cocktail component, release of a manufactured batch, or administration to a patient.

The remaining translational challenge is to connect molecular interpretation with infection-relevant evidence without collapsing these different evidence types into a single claim of therapeutic suitability. Progress will require better-curated phage databases, strain-resolved validation datasets, standardized phenotypic testing, formulation and product-quality studies, PK/PD assessment, and appropriately designed animal and clinical investigations [49].

## 4. Current Bioinformatics Practice and Genomic Evidence in Phage Development

Contemporary therapeutic-phage bioinformatics involves multiple analytical tasks, including viral-sequence identification, genome-quality assessment, gene prediction and annotation, lifestyle classification, comparative genomics, host prediction, receptor and antiphage-defence analysis, and artificial-intelligence-assisted prioritization. These analyses are implemented using tools and databases that differ in their input requirements, reference data, taxonomic resolution, model assumptions, and expected outputs. The evidentiary meaning of a computational result therefore depends not only on the analytical task, but also on the tool used, the quality and representativeness of the input data, the software and database versions, and the biological context in which the output is interpreted.

This section anchors the proposed validation framework in current bioinformatics practice by linking representative tools and analytical strategies to their principal outputs, failure modes, validation requirements, and supported development decisions. The purpose is not to provide an exhaustive software benchmark or recommend a single universal workflow. Rather, it is to clarify how outputs from commonly used approaches can be incorporated into a traceable therapeutic-phage development pathway and where additional experimental or product-level evidence is required.

### 4.1. Representative Bioinformatics Tools and Their Translational Interpretation

Representative bioinformatics tools address distinct stages of therapeutic-phage evaluation, including viral identification, genome-quality assessment, annotation, comparative genomics, lifestyle classification, host prediction, receptor analysis, and bacterial antiphage-defence profiling. Table 3 provides practical examples of commonly used approaches and summarizes how their outputs should be interpreted within the proposed validation framework. The table is not intended as an exhaustive software benchmark, because tool performance depends on input quality, reference coverage, taxonomic composition, analytical parameters, and the intended application.

### 4.2. Genome Sequencing, Annotation, and Comparative Genomics

Therapeutic-phage genome analysis begins with evaluation of raw-read and assembly quality. Read coverage, assembly consistency, mixed populations, host-derived sequences, and genome termini should be assessed where relevant. Completeness estimates can support this evaluation, but they should be interpreted together with read mapping and assembly review, particularly for divergent phages or candidates recovered from metagenomic data [9]. A genome-quality score alone does not establish that the sequenced material is identical to the phage used in subsequent biological experiments or product development.

Genome annotation should document the gene-prediction method, software and database versions, unresolved or hypothetical proteins, and putative risk-associated genomic features. Automated functional assignments may be uncertain when they depend on distant homology or incompletely curated reference databases. Manual curation is therefore particularly important for lifestyle-associated genes, mobile elements, and annotations related to antimicrobial resistance, virulence, or toxin functions [10,15,75]. Where analytical methods produce conflicting results, the disagreement and the rationale for the final interpretation should be reported rather than concealed.

Comparative genomics can support phage-bank organization, candidate clustering, redundancy assessment, and evaluation of shared or divergent genomic modules [66]. However, genomic similarity does not establish equivalent host range, receptor use, or resistance phenotype, while genomic diversity does not necessarily indicate functional complementarity. Comparative-genomic findings should therefore be linked to host-range testing, receptor-related evidence, and resistance-emergence data before they are used to support cocktail-component selection.

For product traceability, the genomic record should link raw-read data, the assembly version, accession number, candidate identifier, preparation identifier, and, where applicable, batch-level sequence confirmation [76]. This documentation allows the identity of the computationally characterized candidate to be related to the material evaluated experimentally or developed as a therapeutic product. It also permits reinterpretation when annotation databases, classification systems, or analytical methods are updated.

### 4.3. Viral Metagenomics and Candidate Discovery

Viral metagenomics expands access to phage diversity beyond candidates recovered through conventional culture-based isolation [77]. Tools such as VIBRANT [8] and geNomad [60] can identify candidate viral sequences from assembled metagenomic data and provide preliminary taxonomic or functional information. This approach can reveal candidate diversity, ecological distributions, and possible phage–host associations, particularly in microbial communities that are incompletely represented by cultured isolates.

The reliability of metagenomic discovery depends on sequence quality and classification confidence. Short or fragmented contigs, uneven coverage, mixed populations, host-derived regions, plasmid-like sequences, and limited representation in reference databases can affect viral identification and downstream annotation. Candidate sequences should therefore undergo assembly review, read mapping, genome-quality assessment, and evaluation for contamination or incomplete boundaries before being used for biological interpretation [8,9].

For therapeutic development, metagenomic sequences are best treated as candidate hypotheses. They may guide the selection of ecological sources, target hosts, or sequences for subsequent isolation or reconstruction, but they do not define a therapeutic product until a physically identifiable phage has been obtained and linked to a traceable genome record. The resulting candidate must then undergo biological characterization, isolate-level activity testing, genome-informed risk assessment, and product-quality evaluation according to the intended therapeutic format [3].

### 4.4. Host Prediction, Receptor Analysis, and AI-Assisted Prioritization

Host-association prediction can use nucleotide and protein similarity, sequence composition, CRISPR spacer matching, receptor-binding-protein features, network-based methods, and machine-learning models [13]. Integrated tools such as iPHoP [70] combine several of these evidence types to predict likely host taxonomy. However, the resulting outputs represent different levels of evidence. Sequence composition or network-based predictions generally support taxonomic association, whereas CRISPR spacer matches and receptor-related features may provide more biologically interpretable hypotheses about previous interactions or host specificity.

CRISPR spacer matching can indicate that a bacterium or a related lineage has previously encountered a phage, but spacer absence does not exclude susceptibility, and spacer presence does not establish productive infection of the tested isolate. Receptor-binding-protein analysis can similarly support hypotheses about adsorption and host range, but receptor expression, capsule state, surface accessibility, and bacterial physiological conditions can alter infection outcomes [72]. Antiphage-defence predictions generated using approaches such as PADLOC [73] or DefenseFinder [74] may help explain resistance, although the genomic presence of a defence system does not demonstrate that it is expressed or functionally active under the tested conditions.

Artificial-intelligence and machine-learning approaches can integrate high-dimensional genomic features and prioritize phage–host combinations that may not be apparent from individual sequence comparisons [14,78]. Their reported performance nevertheless depends strongly on host-label quality, taxonomic resolution, reference coverage, feature selection, and benchmark design. Random training and test splits may overestimate performance when closely related phages or bacterial lineages occur in both datasets. More informative evaluation requires separation of related sequences, testing on previously unseen lineages, and external validation using independent collections of clinical isolates [18,79,80].

**Figure 2:**
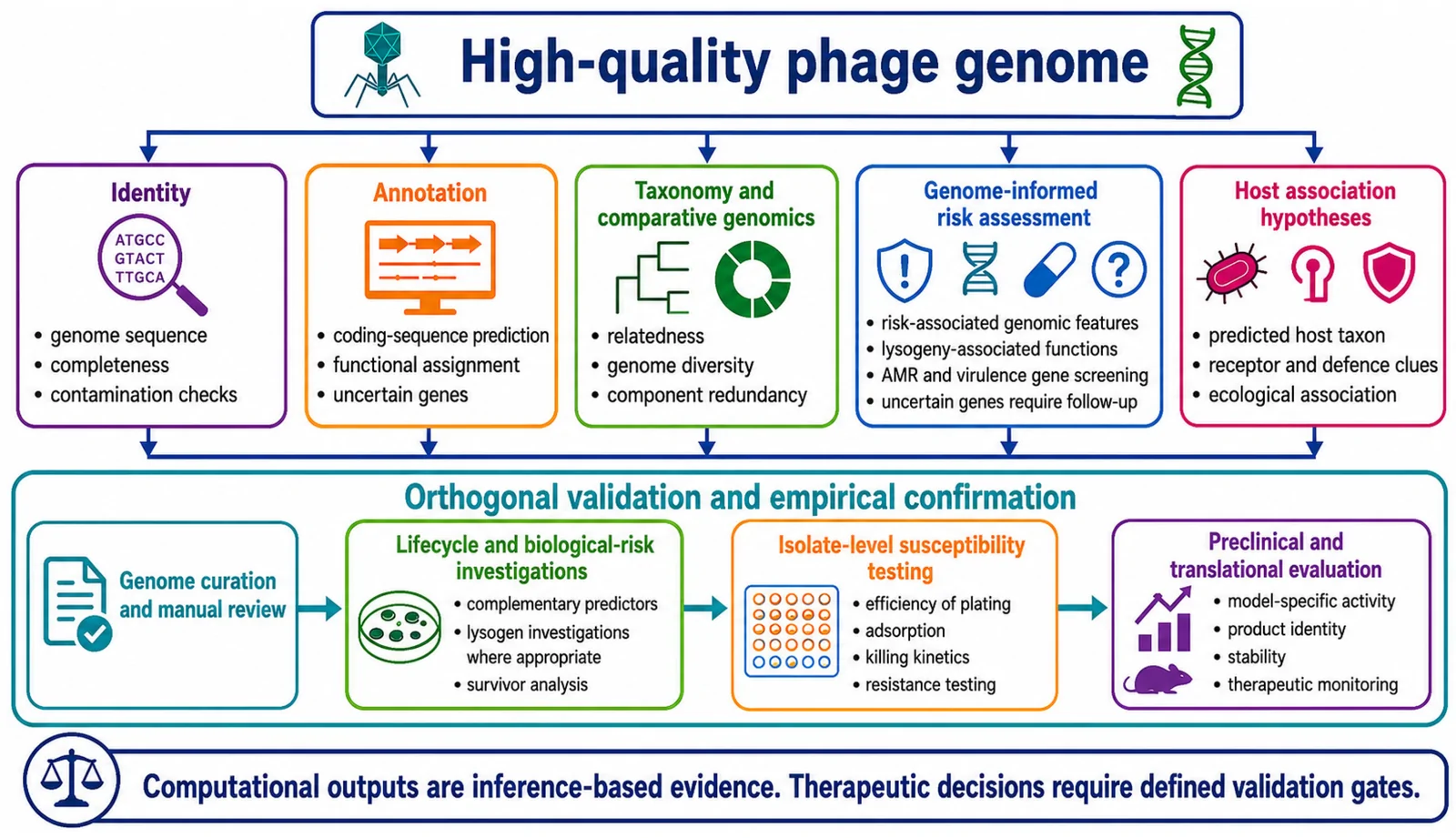
Computational evidence layers and validation gates in therapeutic-phage assessment. Genome-derived evidence can support identity, annotation, taxonomy, genome-informed risk assessment, and host-association hypotheses, but each layer has uncertainty and requires proportionate validation before it can guide a therapeutic decision.

For therapeutic interpretation, ecological association, taxonomic host prediction, receptor-based plausibility, isolate-level susceptibility, and infection-model activity should be reported as distinct evidence levels. Computational outputs can prioritize phage–isolate combinations for testing, whereas therapeutic matching requires phenotypic assays such as efficiency of plating, adsorption, killing kinetics, biofilm activity, and resistance-emergence testing under relevant conditions. Prospective identification of susceptible patient-associated isolates is therefore a more clinically meaningful benchmark than aggregate taxonomic prediction accuracy alone. These evidence levels and their corresponding validation requirements are summarized in Figure 2.

## 5. Iterative Translational Use of Genomic Evidence

Phage-based antibacterial development requires evidence that connects biological activity with product quality and clinical interpretation. Genomics and bioinformatics can support this process by clarifying candidate identity, risk-associated genomic features, host association, and product rationale. However, therapeutic relevance depends on the integration of these computational findings with phenotypic activity, product identity, resistance monitoring, and clinical evidence. This section focuses on those direct genomic interfaces rather than on general formulation or trial-design considerations.

### 5.1. Candidate Selection and Product Characterization

Therapeutic phage selection depends on the convergence of biological, genomic, and pharmaceutical evidence. Genome data can support candidate identity, annotation, taxonomy, and genome-informed risk assessment, but product characterization also requires evidence of activity, purity, potency, stability, and clinical relevance. A genomically suitable candidate should therefore be viewed as an early-stage development component rather than a finished antibacterial product [3].

The evidence required for characterization differs by product format [81]. A live phage product requires information on phage identity, lytic activity, host range, preparation quality, and product-specific risk. Engineered phages require additional attention to design rationale, sequence-confirmed edits, genetic stability, introduced functions, and risk interpretation [34].

Candidate selection is clearest when evidence is separated into layers. The first layer is molecular identity, including genome sequence information. The second layer is biological plausibility, including host association and receptor relevance. The third layer is product quality, including purity, potency, stability, and route suitability. The fourth layer is translational evidence, including PK/PD interpretation, monitoring, and clinical evaluation. These layers should be connected by validation gates and feedback loops rather than treated as independent evidence categories.

### 5.2. Genome-Guided Cocktail Design and Resistance Monitoring

Phage cocktails are often proposed to broaden bacterial coverage and reduce resistance emergence [31]. A host-range matrix can help identify coverage across bacterial isolates, but coverage alone does not establish product robustness. A defensible cocktail rationale should consider component identity, receptor diversity, resistance risk, component compatibility, stability, and relevance to the intended pathogen population [82].

Bioinformatics can support cocktail design by interpreting genome relatedness, receptor-associated features, resistance mechanisms, bacterial strain diversity, and the identity and stability of individual components before and after treatment. Emergent resistance or failed susceptibility testing should trigger bacterial and phage resequencing and a documented decision to retain, replace, or redesign cocktail components. However, it cannot define an optimized cocktail on its own. Empirical host-range testing, interaction assays, resistance monitoring, formulation assessment, and clinical evidence remain necessary before specific outcome claims can be made for phage-combination products [31]. Genome diversity is useful only when it corresponds to demonstrable differences in host range, receptor dependence, or resistance escape.

A rational cocktail should be evaluated as an evolving product rather than a static list of genomes. Component diversity is useful only when it produces complementary receptor use, non-overlapping escape routes, or demonstrated coverage of clinically relevant isolates. Resistance monitoring should specify when a component is retained, replaced, or re-tested, and should distinguish transient phenotypic resistance from stable heritable changes [30,83,84].

### 5.3. Product Identity, Stability, and Therapeutic Monitoring

Product identity and stability are relevant because they connect the sequenced candidate to the material administered to a patient. A genomically suitable phage may be unsuitable for a specific product context if it cannot remain stable during storage, delivery, or administration. Conversely, formulation does not establish target activity. Product quality and therapeutic activity must therefore be evaluated together while retaining their distinct evidentiary roles [50].

Phage PK/PD differs from conventional antibiotic exposure-response relationships because live phages may replicate at the infection site, be cleared by host immunity, vary with local bacterial density, and behave differently across routes of administration and delivery modes [41]. Genomic data can support product comparability and interpretation of sequence drift, but they cannot independently determine exposure, efficacy, or dose.

Therapeutic monitoring may help connect product identity, exposure, immune response, bacterial susceptibility, and clinical outcome [85]. This is especially important for personalized phage use, repeated administration, difficult-to-treat infection sites, and combination strategies. Formulation, stability, PK/PD, and monitoring are integral to moving phage candidates from biological promise toward antibacterial biopharmaceutical development [52]. From a genomic perspective, these pharmaceutical interfaces are most relevant through traceable product identity, sequence stability, and resistance monitoring; genomic evidence does not replace formulation-specific or pharmacological evidence.

## 6. Safety, Quality, and Regulatory Evidence

Safety and quality evidence are central to phage-based antibacterial biopharmaceutical development [51]. Genome analysis can support early candidate review, but it does not remove the need for product-level testing, manufacturing control, pharmacological interpretation, and clinical evaluation [86].

This section considers genome-informed risk assessment, product quality, immune considerations, and regulatory-quality evidence. Bioinformatics can strengthen documentation and risk interpretation, but it should not be treated as independent evidence of product safety or regulatory acceptability [4].

### 6.1. Genome-Informed Risk Assessment and Orthogonal Validation

Genome-informed risk assessment is an important part of candidate triage [86]. It can help identify features that may raise concern, including temperate lifestyle markers, toxin-related annotations, antimicrobial resistance genes, virulence-associated genes, and mobile-element signals. However, the absence of a concerning annotation should not be interpreted as absolute proof of safety, especially because many phage genes remain poorly characterized [10]. This screen therefore identifies potential risks and questions requiring follow-up.

Lifestyle interpretation is particularly important for live phage products [87]. Temperate phages may complicate therapeutic use because lysogeny can influence bacterial phenotype, gene mobility, and product-risk interpretation. Computational tools can support prophage and lifestyle assessment, but their outputs should be interpreted as decision-support evidence rather than definitive conclusions about therapeutic suitability. They should be combined with complementary biological characterization and conservative expert review [33].

Lytic–temperate lifestyle prediction has specific failure modes. Integrase-like genes may be non-functional or insufficient for lysogeny, whereas some integrating phages may use host-encoded recombination functions [88,89]. Clear plaques, phage morphology, and one-step growth curves provide useful supporting evidence but do not independently demonstrate an obligately lytic lifecycle. Consequently, high-quality sequencing and manual annotation should be combined with complementary lifestyle predictors, attempts to detect stable lysogens or inducibility where appropriate, analysis of surviving bacterial isolates, and conservative expert interpretation [17,88,89].

Orthogonal validation should be proportional to the unresolved risk [86]. A candidate with ambiguous integration modules or mobile-element signals warrants more than evaluation using a second in silico classifier [90]. It may require lysogen-recovery attempts, transduction assessment, sequencing of surviving isolates, and product-level impurity testing [90]. Evidence should be recorded as unresolved, supported, or excluded rather than reduced to a binary safe–unsafe classification.

Product-level safety assessment differs by product format [51]. An unmodified lytic phage raises questions about genome identity, lifecycle, host range, and gene-transfer risk. An engineered phage raises additional questions about introduced functions, genome stability, and environmental or regulatory implications. Genome-informed risk assessment should therefore be treated as one layer within a broader product-specific risk assessment that includes preparation purity, potency, and stability.

### 6.2. Product Quality and Immune Considerations

Phage preparations raise quality questions that extend beyond genome content [81]. Product-related concerns include purity, potency, endotoxin level, residual host–cell material, sterility, stability, and batch consistency. These factors determine whether a candidate can be developed as a clinically relevant product, rather than only as a biologically interesting isolate.

In this context, bioinformatics can support traceability and comparability [76]. Genome identity can link the original candidate to subsequent product material, while sequence records can support comparison across batches or development stages. Annotation can also document known risk-associated genomic features. However, these records do not replace product-quality testing and strengthen the evidence package only when they are linked clearly to manufacturing, purity, potency, and stability data [86].

Immune response is also relevant to safety and pharmacology [59]. Phage-specific antibodies, immune clearance, repeat administration, and inflammatory context may influence exposure and clinical outcomes. These considerations are especially important for live phage products, personalized therapy, repeated dosing, and difficult infection sites. The major safety, quality, and regulatory evidence domains for therapeutic phages are summarized in Table 4.

### 6.3. Regulatory-Quality Evidence and Governance

Regulatory-quality evidence remains an evolving area for therapeutic phage products [94]. Classification can differ by jurisdiction and product format, particularly for personalized phage preparations, engineered phages, and adaptable phage-library models. This creates uncertainty regarding how identity, comparability, quality, risk, efficacy, and product-format evidence should be assembled [95].

Personalized phage therapy illustrates the inherent conflict between clinical need and standardized product evidence [96]. It may be valuable when conventional options are limited, but variable product composition complicates documentation, comparability, manufacturing and clinical interpretation. Engineered phages introduce additional governance considerations, including genetic stability, introduced functions, environmental considerations, and product identity [54].

For this review, regulatory-quality bioinformatics is best understood as a tool for evidence documentation and traceability. It can support genome-informed risk assessment, comparability, and transparent reporting, but it cannot replace formal regulatory assessment or product-quality testing [4].

Sequence-resolved documentation also has implications for intellectual property and product governance. Versioned genome records can establish provenance, distinguish parental phages from engineered constructs, and link claimed sequence modifications to the material evaluated experimentally or manufactured. Patentability remains jurisdiction- and claim-specific; engineered phages may be considered through claims directed to defined genomic modifications, technical production methods, formulations, combinations, or therapeutic uses, while biological material and microbiological processes are assessed under the applicable biotechnology-patent framework. Where nucleotide or amino acid sequences are disclosed in a patent application, sequence listings should comply with WIPO Standard ST.26. Bioinformatics can therefore support sequence comparison, novelty assessment, claim traceability, and documentation of engineered changes, but it does not replace formal patentability or freedom-to-operate analysis [97,98].

## 7. Current Challenges and Strategic Priorities

Phage-based antibacterial development is advancing quickly, but several scientific, computational, and translational challenges remain. The field can now draw on stronger sequencing capacity, improved annotation tools, better host-prediction models, and growing clinical experience. However, these advances have not yet resolved the key evidence gaps that affect therapeutic interpretation [3]. A central challenge is that these advances remain insufficiently integrated across discovery, product characterization, and clinical translation. Phage genomics, host prediction, phenotypic testing, product quality, formulation, PK/PD, and clinical evidence are often discussed separately, although they must ultimately support the same development decision. Future progress will depend on linking these evidence types within a coherent and traceable development framework.

### 7.1. Scientific and Methodological Challenges

A major scientific challenge is incomplete phage annotation. Many phage proteins remain hypothetical or poorly characterized, which limits confidence in mechanistic interpretation, genome-informed risk assessment, and product comparison [10]. AI-assisted annotation may improve functional inference, but it cannot overcome incomplete or uneven reference databases [75]. It also cannot substitute for high-quality assemblies or infection-relevant functional evidence, including transcriptomic data where appropriate.

Host prediction is another important limitation. Current computational models can support discovery and prioritization, but strain-level therapeutic matching remains difficult [80]. A model that predicts bacterial genus or species may be useful for ecological interpretation, but this is insufficient for patient-isolate matching. Therapeutic development requires predictions that are validated against clinically relevant isolates under infection-relevant conditions [13].

Bacterial antiphage defense systems add further complexity. Receptor availability, restriction-modification systems, CRISPR-Cas activity, abortive infection, and other defense mechanisms can alter phage susceptibility [99]. These factors explain why phage-host compatibility cannot be inferred from genome similarity alone. Future datasets should integrate phage genomes, bacterial genomes, standardized susceptibility measurements, defence-system profiles, and relevant clinical metadata [80].

### 7.2. Translational and Implementation Challenges

Translational evidence remains uneven. Compassionate-use reports and early clinical studies support feasibility and continued investigation, but they do not establish consistent effectiveness across indications [24]. Clinical interpretation depends on pathogen susceptibility, infection site, concomitant therapy, immune status, route of administration, product quality, and outcome measures [100].

Implementation also differs between fixed and personalized phage models [33]. Fixed products may fit more conventional development pathways, but they require evidence that coverage remains relevant to target pathogen populations. Personalized preparations can support precision-oriented use, but they create challenges in documentation, comparability, manufacturing timing, and clinical interpretation [101].

Improved interdisciplinary communication and shared evidence standards will be essential for therapeutic phage development. Computational scientists need clinically relevant datasets. Clinicians need reports that distinguish prediction from validated activity. Pharmaceutical scientists need traceable product-quality evidence. Regulators and reviewers need clear documentation of product identity, genome-informed risk assessment, quality attributes, and evidence limitations.

### 7.3. Strategic Priorities for Therapeutic Phage Development

Future work should prioritize stronger benchmark datasets, improved functional annotation and closer integration of genomic, phenotypic, pharmaceutical, and clinical evidence. Computational workflows should also remain transparent and reproducible so that analytical outputs can be traced, compared, and re-evaluated as databases and methods evolve [10].

Phage cocktail design and engineered phages require stronger product-specific evidence [3]. Cocktail coverage should be linked to component compatibility, resistance prevention, stability, and relevance to the intended pathogen population. Engineering studies should report the intended genomic change, sequence confirmation, genetic stability, and functional validation rather than treating engineering as a generic enhancement.

Future engineering studies should also report negative and null findings. Failed edits, loss of activity, escape variants, growth-rate penalties, and instability during serial passage are essential for determining whether an engineered construct provides a reproducible advantage over its parental phage. Such reporting would make synthetic-biology claims more comparable and reduce publication bias toward successful constructs [34,55,56].

The strongest future studies will connect genome-resolved candidate identity with standardized phenotypic testing, formulation and stability evidence, PK/PD interpretation, immune monitoring, and clinical outcomes. This combined evidence package is needed to move phage genomics beyond discovery and towards evidence-driven antibacterial biopharmaceutical development. The principal field-level challenges and strategic priorities are summarized in Table 5.

## 8. Minimum Reporting Framework for Genome-Informed Therapeutic Phage Studies

Transparent reporting is particularly important because genomic and computational outputs can represent different levels of evidence. A predicted host association, lifestyle classification, or risk-associated genomic feature should not be presented in the same way as an experimentally validated finding. Studies should therefore state the analytical method used, the quality and limitations of the underlying sequence, the uncertainty of the output, and whether the interpretation was supported by orthogonal or experimental evidence [10,75].

The proposed framework is intended to extend, rather than replace, established minimum information standards. The Minimum Information about an Uncultivated Virus Genome (MIUViG) standard, developed within the Genomic Standards Consortium’s MIxS framework, specifies reporting of virus origin, sequence-generation and identification methods, assembly quality, genome completeness, annotation, taxonomic classification, and in silico host prediction [102]. These elements correspond to the sequencing, assembly, annotation, and host-association domains in Table 6. Therapeutic-phage studies additionally require product-linked information not covered by MIUViG, including isolate-level susceptibility, linkage between the sequence and the tested or administered preparation, batch identity, stability, engineered modifications, and monitoring or redesign criteria.

Reporting should also allow the evidence pathway to be traced from the sequenced candidate to the tested or administered product. Sequence accession numbers and versions should be linked to candidate identifiers, preparation records, and relevant batches. For cocktails, personalized preparations, and engineered phages, the identity and validation status of each component or genomic modification should be documented clearly [76,81].

Studies should not only report positive findings, but also unresolved annotations, conflicting predictions, failed validation, resistance emergence, and criteria for candidate replacement or redesign. This prevents uncertainty from being concealed and allows subsequent investigators, clinicians, and regulators to determine which conclusions are supported, which remain provisional, and which require further investigation. Table 6 therefore provides a practical minimum reporting framework for genome-informed therapeutic phage studies.

## 9. Conclusions

Phage genomics and bioinformatics provide decision-support evidence for therapeutic-phage development. They can establish or refine candidate identity, support annotation and comparative analysis, identify risk-associated genomic features, and generate testable hypotheses about host compatibility, resistance, and cocktail complementarity. Their translational value depends on transparent uncertainty reporting and on the validation performed after each computational output.

Genome-derived outputs are not proof of an obligately lytic lifecycle, isolate-level susceptibility, product purity, potency, stability, pharmacokinetics/pharmacodynamics, safety, or clinical benefit. Each output should therefore pass a defined validation gate, including assembly and annotation review, phenotypic testing against intended isolates, product-quality testing, and infection-relevant or clinical evaluation where appropriate.

The iterative framework proposed here links genome generation, computational inference, phenotypic validation, product decision-making, and therapeutic monitoring. Bacterial resistance, sequence drift, failed susceptibility testing, or product instability should trigger resequencing and documented candidate, cocktail, or engineered-phage redesign. This closed-loop use of genomic evidence can strengthen therapeutic-phage development without overstating what bioinformatics alone can establish.

## Figures and Tables

**Table 1:** Comparison of the principal emphasis of selected recent reviews.

Primary Focus	Genome Analysis and Annotation	Host Prediction, Validation, and Engineering	Key Gap Addressed	Reference
Clinical and translational challenges	Limited emphasis	Broad discussion	Limited emphasis on explicit validation gates	[3]
Clinical therapeutic-phage practice	Candidate screening discussed	Strong phenotypic emphasis; limited engineering and feedback	Limited genomics-to-redesign integration	[17]
Phage annotation and nomenclature	Detailed	Mainly annotation-related; limited engineering and feedback	Limited linkage to validation and product decisions	[10]
Genomics and standardization in phage therapy	Detailed	Host prediction, validation, and engineering discussed	Limited closed-loop redesign framework	[15]
Genomics, AMR detection, and phage therapy	Broad	Broad host-prediction coverage; limited engineering and feedback	Limited distinction between host prediction and isolate-level evidence	[7]
Present review: translational evidence and validation gates	Detailed and linked to traceability	Explicit separation of prediction, isolate-level validation, engineering, and feedback	Iterative framework linking computational evidence to validation, product decisions, monitoring, and redesign	Present review

Note: The comparison reflects the principal emphasis of each publication and does not imply that topics described as having limited emphasis were entirely absent.

**Table 2:** Therapeutic phage formats and principal evidence requirements.

Therapeutic Format	Development Logic	Principal Evidence Required	References
Unmodified lytic phage	Single phage selected against a susceptible strain	Genome identity, lytic activity, host range, risk assessment, and preparation quality	[28,29]
Fixed phage cocktail	Defined combination intended to broaden coverage	Component identity, combined host range, compatibility, resistance testing, and stability	[30,31]
Personalized phage preparation	Phage selected for a patient-associated isolate	Isolate susceptibility, traceable selection, rapid quality control, and clinical documentation	[32,33]
Engineered phage	Genetically modified phage with altered or added activity	Design rationale, sequence confirmation, stability, activity, and risk assessment	[34,35]
Phage–antibiotic combination	Combined treatment intended to improve activity or limit resistance	Interaction testing, resistance dynamics, dose conditions, and PK/PD interpretation	[36,37]

**Table 3:** Representative bioinformatics tools and their translational interpretation in therapeutic-phage development.

Analytical Task	Representative Tools or Approaches	Principal Output	Key Limitations	Translational Interpretation
Viral identification and genome quality	VIBRANT [8], geNomad [60], CheckV [9], PhageTerm [61]	Viral-sequence identification, genome completeness, contamination, termini, and packaging information	Fragmented assemblies, uncertain boundaries, mixed populations, and reduced accuracy for novel phages	Supports selection of sequences that are sufficiently resolved for further characterization; requires read mapping, assembly review, and confirmation of the intended phage
Gene prediction, annotation, and risk screening	Pharokka [62], PHANOTATE [63], PHROGs [64], and curated AMR or virulence databases	Coding sequences, predicted protein functions, hypothetical proteins, and putative risk-associated features	Database incompleteness, hypothetical proteins, and homology-based overannotation	Supports candidate triage and identification of features requiring manual curation or experimental follow-up
Comparative genomics	VIRIDIC [65] and complementary gene-content or protein-level comparisons [66]	Genome relatedness, candidate clustering, shared modules, and redundancy	Genomically related phages may differ in host range, receptor use, or resistance phenotype	Supports organization of phage collections and preliminary selection of potentially complementary cocktail components
Lytic–temperate lifestyle prediction	BACPHLIP [67], PhaTYP [68], and PhageAI [69]	Predicted virulent or temperate lifestyle	Training-set bias, incomplete genomes, annotation uncertainty, and noncanonical integration mechanisms	Supports candidate prioritization or exclusion only when combined with manual annotation and lysogeny-oriented testing
Host and receptor prediction	iPHoP [70], CRISPR spacer matching approaches [71], and receptor-binding-protein analysis [72]	Predicted host taxonomy, historical phage–host association, and receptor-use hypotheses	Limited strain-level resolution and context-dependent receptor expression	Supports prioritization of phage–isolate combinations for efficiency-of-plating, adsorption, killing, and resistance testing
Antiphage-defence profiling	PADLOC [73] and DefenseFinder [74]	Predicted bacterial defence systems	Gene presence does not establish expression or functional resistance	Supports interpretation of unexplained resistance and design of genotype-informed susceptibility assays

Note: These lifestyle-prediction tools use different evidence representations. BACPHLIP applies a random-forest classifier to conserved protein domains, PhaTYP uses BERT embeddings of protein-cluster tokens and was developed to improve prediction on short contigs, and PhageAI applies nucleotide-sequence word embeddings with supervised machine learning. Because performance depends on genome completeness, training data, and benchmark design, these outputs should guide rather than replace manual annotation and lysogeny-oriented phenotypic validation.

**Table 4:** Safety, quality, and regulatory evidence requirements for therapeutic phage products.

Evidence Domain	Why It Matters	Required Non-Computational Evidence	References
Lifestyle and lysogeny assessment	Lysogeny may alter bacterial phenotype and facilitate gene mobility	Lysogen-recovery or induction studies, biological characterization, and conservative risk interpretation	[87,90]
AMR, virulence, and toxin screening	Risk-associated genes may affect candidate suitability	Expert curation and product-specific risk assessment	[15,86]
Transduction and mobile elements	Gene-transfer potential affects benefit–risk evaluation	Transduction assays, mobile-element assessment, and sequencing of surviving isolates where appropriate	[86,91]
Purity and residual impurities	Host-derived material may affect safety and product quality	Endotoxin, sterility, residual host-material, purity, and batch-consistency testing	[48,86]
Immune response	Host immunity may influence exposure, repeat dosing, and monitoring	Immunological and pharmacological assessment	[59,85]
Resistance emergence	Bacterial adaptation may reduce therapeutic activity	Host-range testing, resistance monitoring, and activity assays	[84,92]
PK/PD uncertainty	Exposure depends on bacterial density, route, host factors, and infection site	Pharmacological modelling, therapeutic monitoring, and clinical interpretation	[41,93]
Regulatory classification	Product format affects regulatory and evidence requirements	Jurisdiction-specific regulatory and quality assessment	[94,95]

**Table 5:** Current challenges and strategic priorities in therapeutic phage development.

Challenge	Main Limitation	Strategic Priority	References
Incomplete phage annotation	Many proteins remain hypothetical or poorly characterized	Improve curated databases, functional annotation, and uncertainty reporting	[10,75]
Strain-level host prediction	Species-level predictions may overstate therapeutic relevance	Develop benchmarks based on patient isolates and strain-level susceptibility	[79,80]
Antiphage defence complexity	Bacterial defence systems can alter infection outcomes	Integrate defence-system profiling with host-range and susceptibility testing	[84,99]
Cocktail evidence gaps	Broad coverage does not establish component compatibility or robustness	Link cocktail design to compatibility, resistance escape, and stability	[30,31]
Phage–antibiotic variability	Interactions depend on strain, drug, dose, and exposure conditions	Evaluate combinations under clearly defined experimental conditions	[36,83]
Engineered-phage uncertainty	Genetic modification introduces additional activity, stability, and risk questions	Require sequence confirmation, functional validation, and product-specific risk assessment	[34,57]
Bioinformatics traceability	Analytical workflows may lack reproducibility and auditability	Use versioned, transparent, and reproducible computational documentation	[10,76]

**Table 6:** Recommended minimum reporting elements for genome-informed therapeutic phage studies.

Reporting Domain	Minimum Information to Report	Validation or Status to State	References
Source, sequencing, and identity	Sample or environmental source, isolation or reconstruction status, viral-identification method, sequencing platform, read type, accession number, sequence version, and candidate identifier	Whether the sequence represents an uncultivated viral genome, cultured isolate, or reconstructed candidate, and whether the tested or administered material is sequence-traceable	[10,76]
Assembly quality	Assembler and version, read-mapping support, completeness, termini where relevant, and contamination or mixed-population assessment	Whether the genome is sufficiently resolved for downstream interpretation	[9,10]
Annotation	Annotation software, database and version, uncertain or hypothetical proteins, and screening for risk-associated genomic features	Which annotations remain uncertain and require further investigation	[10,15,75]
Lifestyle interpretation	Methods used, supporting evidence, limitations, and any lysogeny-oriented follow-up	Whether the lifecycle interpretation remains computational or is supported by complementary evidence	[87,88,89]
Host association	Prediction method, taxonomic resolution, receptor or defence evidence, and intended bacterial population	Whether the output represents ecological association, taxonomic prediction, mechanistic plausibility, or isolate-level evidence	[18,79,80]
Isolate susceptibility	Bacterial-isolate metadata, efficiency of plating, adsorption, killing kinetics, biofilm testing, and resistance-emergence methods where relevant	Whether activity was demonstrated against the intended isolates and experimental conditions	[17,49,88]
Engineered phages	Intended genomic modification, sequence confirmation, construct stability, and tests for activity, escape, and unintended effects	Whether product-specific validation supports the intended engineered function	[34,35,57]
Product identity and stability	Linkage between the sequenced candidate and product material, batch comparability, stability, purity, and potency evidence	Whether product-level evidence supports use of the characterized candidate	[76,81]
Therapeutic monitoring	Bacterial and phage resequencing plans, susceptibility monitoring, resistance criteria, and redesign triggers	How new evidence will inform candidate retention, replacement, or redesign	[84,85,93]

Note: This framework is not intended to replace product-specific regulatory guidance. Reporting requirements will vary according to the development stage, therapeutic format, intended indication, and jurisdiction. Nevertheless, consistent reporting across these domains would make computational claims easier to evaluate and would strengthen the connection between genome analysis, experimental validation, product characterization, and translational decision-making. The genome-reporting fields are aligned conceptually with MIUViG and the broader MIxS framework, whereas the product-linked fields extend beyond these standards to address therapeutic development.

## Data Availability

No new data were created or analyzed in this study. Data sharing is not applicable to this article.
